# Environmental pollution and cancer

**DOI:** 10.1016/j.jped.2024.09.004

**Published:** 2024-10-30

**Authors:** Denise Bousfield da Silva, Mara Albonei Dudeque Pianovski, Neviçolino Pereira de Carvalho Filho

**Affiliations:** a Departamento de Pediatria, Universidade Federal de Santa Catarina, Florianópolis, Santa Catarina, Brazil; b Departamento de Pediatria, Universidade Federal do Paraná, Curitiba, Paraná, Brazil; c Departamento de Oncologia Pediátrica, Casa de Saúde Santa Marcelina, São Paulo, São Paulo, Brazil

**Keywords:** Environmental pollutants, Occupational cancer, Prevention, Soil pollutants

## Abstract

**Objective:**

To identify and describe pollutants with carcinogenic potential that contaminate indoor and outdoor air, food and soil.

**Data source:**

The descriptors environmental pollutants, occupational cancer, prevention and soil pollutants were used to conduct the research for literature review. Articles published from 2003 to 2024 in the electronic databases Pubmed Medline, Lilacs and Scielo, in Portuguese and English, were included.

**Summary of findings:**

There are multiple sources of pollution in the external and internal environments, including motor vehicles, industrial facilities, smoke from tobacco products, agricultural activities, fires and domestic combustion devices. The most important pollutants related to chemical substances include all forms of asbestos, benzene, exhaust gases from gasoline engines, food and water contaminants, such as arsenic and inorganic arsenic compounds, in addition to persistent organic pollutants, such as dioxins. The use of fossil fuels and biomass for domestic heating are also important sources of pollution. The carcinogenic potential of pollutants varies according to the sources of pollution, climate conditions and the region's topography.

**Conclusions:**

Global environmental pollution is an international public health problem with multiple health effects. Many environmental pollutants are proven to be carcinogenic to adults, while few causes have been scientifically established for children. Pollution is mainly caused by uncontrolled urbanization and industrialization. Preventing environmental exposure to carcinogenic pollutants requires both government regulation and community action and commitment.

## Introduction

The impact of cancer in the world in 2020, based on estimates from the Global Cancer Observatory (GCO) and prepared by the International Agency for Research on Cancer (IARC), indicates that there were 19.3 million new cases of cancer worldwide

In men, lung cancer is the most frequent one, followed by prostate, colon and rectal, non-melanoma skin and stomach cancer. In women, breast cancer is the most common, followed by colon and rectal, lung, cervical and non-melanoma skin cancer.^1^

In Brazil, the National Cancer Institute (INCA, *Instituto Nacional do Câncer*) estimates that there will be 704,000 new cases of cancer per year for the three-year period from 2023 to 2025, and 483,000, if non-melanoma skin cancer cases are excluded. This is estimated to be the most common type of cancer (31.3%), followed by breast (10.5%), prostate (10.2%), colon and rectum (6.5%), lung (4.6%) and stomach cancer (3.1%).^1^

INCA estimated that for each year of the 2023/2025 triennium, there will be 7,930 new cases of cancer among children and adolescents in the country. In the age group of one to 19 years, cancer is the second leading cause of death in Brazil.^1^

The causes of childhood cancer are still unknown, with at least 5% being hereditary. Ionizing radiation is the only confirmed environmental carcinogen in this age group.^1^ In adults, however, cancer is associated with aging and long-term exposure to carcinogens.^2^ In this context, it is important for children to receive guidance on preventing cancer in adulthood.

There are thousands of natural or man-made substances present in the environment, including in the workplace, that can cause cancer. There are multiple sources of risk, including both indoor and outdoor environments, from motor vehicles, industrial facilities, and tobacco smoke to agricultural activities, fires, and household combustion devices.^2^^,^^3^

The Global Burden of Disease study estimated that in 2019, 50.6% of cancer deaths in men and 36.3% in women were attributable to behavioral, environmental, occupational, or metabolic risk factors.^4^

## Environmental pollution

“Environment” is typically defined as the set of physical, chemical, and biological conditions external to the human host, in addition to all conditions related to behaviors.^3^

Air pollution originates from several emission sources, both natural and anthropogenic, with the latter being dominant since the beginning of industrialization. The combustion process is the largest contributor to air pollution, particularly from fossil fuels and biomass used to generate energy.^5^

In indoor environments, unventilated heating fuels, cooking stoves, tobacco combustion and combustion for other purposes are important sources of pollution.^6^

Pollutants associated with solid fuel in indoor environments mainly include polycyclic aromatic hydrocarbons, particulate matter, nitrous oxide, carbon monoxide and sulfur dioxide. These pollutants can lead to a variety of health risks, inducing different toxicity mechanisms, such as oxidative stress, DNA methylation and gene activation.^6^

The use of fossil fuels and biomass for domestic heating are also important sources of pollution. External combustion sources include air, soil, water, industry, power generation and biomass burning, which includes forest and savanna fires, the burning of agricultural residues, as well as the burning of waste in urban areas. Other sources that contribute to external pollution include dust resuspension and construction activities.^5^

Some of the chemicals present in the environment that are the most important pollutants contaminating indoor and outdoor air include all forms of asbestos, benzene, exhaust gases from gasoline engines, food and water contaminants such as arsenic and inorganic arsenic compounds, and persistent organic pollutants such as dioxins.^2^

The carcinogenic potential of pollutants varies according to the pollution sources, climate conditions and region topography. In 2021, the World Health Organization (WHO) estimated that each year, 7 million premature deaths occur worldwide due to air pollution. Measures to reduce these levels can reduce the burden of heart disease, respiratory disease, and cancer, especially lung and bladder cancer.^7^

Regarding ambient air, George et al. evaluated 172,550 patients (aged 1–19) selected from the National Cancer Database to investigate how living in areas with high air pollution affects the overall survival of children with cancer in the United States of America. The study concluded that living in areas with high pollution is associated with worse overall survival. This finding emphasizes the need for stricter air quality standards to better protect children, particularly those with serious health problems such as childhood cancer.^8^

Global air pollution is an international public health problem with multiple health effects, particularly for susceptible groups, such as children, who are vulnerable during their development. In relation to childhood cancer, it is necessary to conduct genotoxicity and carcinogenicity studies, in addition to evaluating other potential biological effects, including epigenetic modifications.^9^

Considering the scope and complexity of the subject, this article will address some of the main environmental carcinogens for humans. Many environmental pollutants that will be addressed are proven to be carcinogenic for the adult population, while few causes have been scientifically established for children.

## Smoking

Smoking is the predominant risk factor in most studies that evaluate the epidemiology of lung cancer.^10^ Worldwide, it is estimated that 2.4 million deaths per year are related to cancer caused by tobacco. Without dramatic declines in tobacco use, tobacco products are expected to cause one billion deaths worldwide in this century, mainly in low- and middle-income countries.^2^

Estimates of the relative risk of lung cancer in long-term smokers compared with non-smokers range from 10- to 30-fold.^11^

The risk of lung cancer increases with the number of cigarettes smoked per day and the duration of smoking over one's lifetime. Other factors that may influence smoking include age at the start, the degree of inhalation, tar content, the nicotine content of cigarettes, and use of unfiltered cigarettes. Additionally, it is important to consider that reducing or stopping smoking gradually reduces the risk of lung cancer, although not to baseline levels.^11^

In most countries, it is estimated that 15–50% of the population is exposed to secondhand smoke.^2^ Secondhand smoke inhalation is also a significant risk factor for lung cancer, and in geographic regions with limited resources, there are other additional factors that may contribute, such as smoke and air pollution.^10^

Smoking is also implicated as a causal factor for leukemia, as well as for cancers of the oral cavity, nasal cavity, paranasal sinuses, nasopharynx, larynx, esophagus, pancreas, liver, stomach, cervix, kidney, large intestine and bladder. Some studies also suggest that smoking is associated with an increased incidence of breast and prostate cancer, particularly in African-Americans.^12^^,^^13^

Another risk factor for cancer that remains to be determined is the impact of the use of electronic nicotine devices and other emerging products, as well as the dual use. Laboratory studies can currently measure the carcinogens in these new exposure products among users, but it is not yet possible to determine the potential long-term effects on cancer risk or the impact of cigarette smokers becoming dual users. An emerging concern is that young people may become addicted to nicotine via electronic devices and then switch to cigarette smoking.^2^

Cigar or pipe smoking is also associated with an increased risk of lung cancer.^14^

## Opium, Marijuana, and Cocaine

In the Galeston cohort study with 50,045 participants in Iran, opium use was associated with a dose-dependent increased risk of developing lung cancer (hazard ratio 2.2).^15^

The risk of lung cancer from smoking marijuana or cocaine is less clear than that from smoking tobacco. An association between lung cancer and smoking these agents has been difficult to prove because studies have been limited by selection bias, small sample sizes, and failure to adjust to tobacco smoking. However, the possibility of adverse effects from the consumption of these agents cannot be excluded.^16^

## Asbestos

There is a clear association between occupational exposure to asbestos and the subsequent development of lung cancer, with the risk being dose-dependent and varying according to the type of asbestos fiber. The degree to which low-level non-occupational exposure to asbestos increases the risk of lung cancer is less well defined. However, the potential risk is a major public health concern due to the large number of individuals who work or attend school in buildings containing asbestos, and the cost and potential hazards of asbestos removal.^2^

Most cases of mesothelioma occur due to occupational exposure to asbestos in activities such as mining, milling, and bagging asbestos, as well as the industrial manufacturing of materials composed of the substance. It is estimated that 4% to 20% of mesothelioma cases can be attributable to environmental exposure to asbestos.^2^^,^^7^

## Radon

Uranium miners who have been occupationally exposed to radon and its decay products have an increased risk of lung cancer, and an interactive effect has been observed between radon exposure and smoking.^17^

Radon is present in soil, rocks, and groundwater and can accumulate in homes. A 2005 meta-analysis of 13 European case-control studies reported a linear relationship between the amount of radon detected in the home and the risk of developing lung cancer. Based on this meta-analysis, the authors estimated that radon exposure may be responsible for approximately 9% of lung cancer deaths and 2% of all cancer deaths in Europe.^18^

## Cooking and heating emissions

The burning of biomass fuels (e.g., wood, animal dung, coal, plants) produces pollutants such as carbon monoxide, nitrogen dioxide, formaldehyde, benzene, polyaromatic hydrocarbons, and other particulates. Grilling over charcoal or using wood-burning stoves at home or in restaurants are examples of this risk. The reported health effects of heating and cooking emissions include asthma and other respiratory symptoms, increased risk of respiratory tract infections including tuberculosis, sensitization to aeroallergens, and cancers such as lung, cervical, and upper aerodigestive tract cancers.^19^

The importance of indoor air pollution as a cause of lung cancer is illustrated by a retrospective cohort study involving over 27,000 individuals from China. Lifetime burning of bituminous coal, associated with smoke, was associated with a significantly increased incidence of lung cancer compared with those who used anthracite (smokeless) coal, with a risk ratio of 36 for men and 99 for women. The lifetime risk of developing lung cancer was approximately 20% for men and women who used bituminous coal, compared with 0.5% for those who used anthracite coal.^20^

## Diesel engine exhaust

Outdoor air pollution is a complex mixture of pollutants originating primarily from fuel used in transportation, power generation, industrial activity, biomass combustion, and domestic heating and cooking. Several agents or mixtures have been established as carcinogens to humans, including benzene, 1,3-butadiene, diesel engine exhaust, silica dust, benzopyrene, chromium, arsenic, and asbestos.^2^

In 2017, air pollution from several sources, including diesel engine exhaust and industrial processes, independently accounted for an estimated 350,167,000 lung cancer deaths worldwide.^2^^,^^7^

A prospective analysis of data from the European Cohort Study of Air Pollution Effects using records from 17 cohort studies from nine European countries, involving more than 300,000 participants, found a significant association between air pollution from particulate matter and lung cancer incidence. This risk was proportional to the extent of exposure and persisted even after adjustment for any confounding effects of smoking.^20^

## Arsenic

Long-term exposure to high levels of arsenic in drinking water is associated with an increased risk of certain cancers, with strong evidence supporting a dose-response relationship for bladder cancer.^21^

The evidence linking arsenic in drinking water with the risk of lung, skin, and bladder cancer comes primarily from populations in areas with very high natural arsenic levels, including Argentina, Bangladesh, northern Chile, West Bengal in India, and Taiwan in China. The average arsenic exposure varies, and areas considered to have high arsenic levels are those with concentrations above 100 μg/L.^2^^,^^22^

## Erionite and fluoro-edenite

Erionite, because of its fibrous structure, has been shown to cause mesothelioma in studies carried out in Turkey. This finding was also confirmed by another study carried out in Mexico. The most recent findings regarding mesothelioma have been associated with fluoro-edenite, an amphibole fiber.^2^

## Chlorination byproducts in drinking water

Chlorination of drinking water is used for disinfection, and during this process, chlorine reacts with the organic matter in the water to produce a mixture of byproducts. Recent studies in individuals exposed to disinfection byproducts have identified novel biological pathways and genomic responses indicative of increased cancer risk. Chlorination byproducts in drinking water have been consistently associated with bladder cancer risk.^2^

## Nitrates

Exposure to nitrates in drinking water has been examined in case-control and cohort studies in relation to several types of cancer, including stomach, esophageal, brain, bladder, breast, colorectal cancer, and lymphomas. The IARC, in its monographs, concluded that there is inadequate evidence in humans for the carcinogenicity of nitrate in drinking water, but that nitrate or nitrite ingested under conditions resulting in endogenous nitrosation is probably carcinogenic to humans (Group 2A).^2^

## Heavy metals

Heavy metals dispersed in the environment as a result of their industrial use can contaminate water, soil and air. Non-essential metals (aluminum, nickel, thallium, lead, cadmium, mercury and beryllium) are highly toxic, even in small concentrations, and are also considered contaminants for the ecosystem due to their characteristics of persistence in the environment, bioaccumulation and high toxicity.^23^

According to IARC, heavy metals such as cadmium, lead and chromium (Table 1) are particles considered carcinogenic to humans.^2^^,^^3^

**Table 1:** Some environmental pollutants evaluated as carcinogenic to humans and classification, according to the monographs of the International Agency for Research on Cancer (IARC).

Agent	Location/histological type of cancer	IARC monograph classification
Outdoor air pollution		
Outdoor air pollution, pollution/particulate matter in outdoor air	Lung	Group 1
Diesel engine exhaust, silica dust, benzene (mainly occupational)	Lung, leukemias, lymphomas	Group 1
Indoor air pollution		
Indoor emissions from household combustion of coal	Lung	Group 1
Indoor emissions from household combustion of biomass fuel (mainly wood)	Lung	Group 2A
Passive smoking	Lung	Group 1
Smoking	Lung, bladder, kidney, cervix, liver, nasal cavity, paranasal sinuses, some types of leukemia and lymphomas, nasopharynx, esophagus, stomach, ovary	Group 1
Other pollutants - benzene, 1,3-butadiene, diesel engine exhaust, ethylene oxide, formaldehyde, polychlorinated biphenyls (mainly occupational)	Lung, leukemias, lymphomas, nasopharynx and others	Group 1
Asbestos and other fibers		
Asbestos	Lung, mesothelioma, larynx, ovary	Group 1
Erionite, fluoro-edenite	Mesothelioma	Group 1
Drinking water contaminants		
Arsenic	Lung, skin, bladder	Group 1
Nitrates	Stomach	Group 2A
Soil and food contaminants, including pesticides		
Dioxin (2,3,7,8-tetrachlorodibenzo-para-dioxin)	All neoplasias	Group 1
Polychlorinated biphenyls	Skin, melanoma	Group 1
Lindane	Lymphomas	Group 1
Several other pesticides	Mainly leukemias and lymphomas	Group 2A
Metals in water and soil		
Cadmium, lead, chrome	Lung	Group 1
Ionizing and ultraviolet radiation		
Radon-222 and its decay products (indoor air)	Lung	Group 1
Solar radiation	Skin, melanoma	Group 1
Tanning devices that emit ultraviolet radiation	Cutaneous and ocular melanoma	Group 1
X-ray and gamma radiation	Bone, bladder, central nervous system, breast, colon, rectum, lung, esophagus, salivary gland, skin, kidney, stomach, thyroid	Group 1

Source: adapted from International Agency for Research on Cancer^2^ and Espina et al.^3^

The harmful effects on health caused by environmental exposures usually depend on the agent's carcinogenic potential, the dose, duration and intensity of exposure, in addition to individual susceptibility.^3^ Based on IARC monographs, Table 1 describes the classification of general outdoor air pollution into particles considered carcinogenic to humans (group 1) and probably carcinogenic (group 2A).^2^

The specific types of cancer associated with occupational exposures are mainly lung, skin (non-melanoma), bladder cancer and mesothelioma.^24^

Currently, approximately 10.8% of cancer cases (excluding non-melanoma skin cancer) in men and 2.2% in women are caused by occupational exposure.^24^ Therefore, pediatricians should remember to investigate the parents’ history of environmental or occupational exposures, considering that they may increase the risk of cancer for their offspring.^3^

The main types of cancer-related to occupational exposure to metals are listed in Table 2.^23^^,^^24^

**Table 2:** Work-related malignant neoplasms, according to etiological agents or risk factors.

Disease	Etiological agents or risk factors of occupational nature
Malignant stomach neoplasm	Asbestos
Malignant larynx neoplasm	Asbestos
Mesothelioma	Asbestos
Malignant neoplasm of the bronchi and lungs	Asbestos, arsenic and arsenical compounds, beryllium, nickel, cadmium or its compounds, chromium and its toxic compounds, vinyl chloride, chloromethyl ethers, free silica, tar, pitch, bitumen, mineral coal, paraffin and waste products of these substances, ionizing radiation, coke oven emissions, nickel and its compounds, acrylonitrile, aluminum industry (foundries), mineral cutting oils, metal foundries
Liver angiosarcoma	Arsenic and its arsenical compounds, vinyl chloride
Other malignant skin neoplasms	Arsenic and its arsenical compounds, tar, pitch, bitumen, mineral coal, paraffin and waste products of these substances causing skin epitheliomas, ionizing radiation, ultraviolet radiation
Malignant pancreatic neoplasm	Vinyl chloride, epichlorohydrin, aliphatic and aromatic hydrocarbons in the petroleum industry, cadmium
Malignant neoplasm of the nasal cavity and paranasal sinuses	Ionizing radiation, nickel and its compounds, wood dust and other organic dust from the furniture industry, dust from the leather industry, organic dust (in the textile industry and in bakeries), petroleum industry
Malignant neoplasm of bones and articular cartilage of the limbs	Ionizing radiation
Leukemias	Benzene, ionizing radiation, ethylene oxide, antineoplastic agents, electromagnetic fields, chlorinated pesticides
Malignant neoplasm of the bladder	Tar, pitch, bitumen, mineral coal, paraffin and waste products of these substances, aromatic amines and their derivatives, emissions of coke ovens, arsenic, cadmium
Malignant kidney neoplasm	Arsenic
Malignant prostate neoplasm	Arsenic, cadmium
Malignant breast neoplasm	Cadmium
Malignant neoplasm of the endometrium	Cadmium

Source: adapted from Instituto Nacional de Câncer^23^ and Instituto Nacional de Câncer José Alencar Gomes da Silva.^24^

## Ionizing and non-ionizing radiation

Radiation is the emission and propagation of energy through space or a material medium in the form of electromagnetic waves or subatomic particles. There are several types of radiation, which are generally categorized as ionizing and non-ionizing, depending on their ability to ionize atoms and molecules.^25^

Radiation sources can be natural or artificial. Natural sources include cosmic radiation, terrestrial radiation from radioactive materials in the soil, and radon in the air. Examples of artificial sources include medical and dental equipment (X-rays, radiotherapy), nuclear reactors, nuclear weapon testing, and some industrial applications.^25^

Radiation exposure can have a variety of health effects, depending on the type and dose of radiation. Ionizing radiation is a type of radiation that has enough energy to remove electrons from atoms or molecules, ionizing them. This ionizing ability can cause damage to DNA and other cell structures, which can lead to mutations and cancer. Common sources include X-rays, gamma radiation, cosmic radiation, and natural and artificial radioactive materials.^26^

Acute exposure to high doses of ionizing radiation can cause burns, acute radiation sickness, and an increased risk of cancer. Chronic exposure to low doses, such as that from repeated medical examinations, can also increase the risk of cancer in the long term.^26^

Epidemiological studies have consistently shown an increased risk of cancer in children exposed to ionizing radiation. Children are particularly sensitive to the effects of radiation because of their rapid cell growth and division.^27^

Medical exposures to ionizing radiation, such as radiotherapy and diagnostic imaging, are also significant sources of risk. Radiotherapy, although essential in the treatment of many types of cancer, can induce subsequent malignancies.^27^

Moderate to high doses of radiation are well-established causes of cancer, especially when exposure occurs at a young age.^27^ Abalo et al. conducted a literature review (meta-analysis) on the cancer risks associated with pre- and postnatal exposure to ionizing radiation for medical diagnosis in children and concluded that exposure to computed tomography (CT) in childhood seems to be associated with an increased risk of cancer, especially leukemia and brain tumors, while no significant association was observed with diagnostic radiography.^28^

Bosch de Basea Gomez et al. found compatible results when following and evaluating 948,174 individuals submitted to CT examinations before the age of 22 in nine European countries. The results suggest that for every 10,000 children (mean dose of 8 mGy), 1–2 people are expected to develop a hematologic malignancy attributable to radiation exposure in the subsequent twelve years. They reinforce that there is evidence of an increased risk of cancer at low doses of radiation and highlight the ongoing need for pediatricians to assess the precise indication of CT examinations and dose optimization.^29^

A study conducted in Switzerland evaluated the relationship between the incidence of childhood cancer and levels of exposure to external environmental radiation from terrestrial and cosmic gamma rays using data from a cohort study based on a national census. Of 3,401,113 children followed, 3,137 cases of cancer were identified, including 951 leukemias, 495 lymphomas, and 701 cases of central nervous system (CNS) tumors. The hazard ratios per 1 mSv (thousandths of a sievert) increase in cumulative external background radiation dose were 1.04 (95% CI: 1.01–1.06) for all combined cancers, 1.06 (1.01–1.10) for leukemia, 1.03 (0.98–1.08) for lymphoma, and 1.06 (1.01–1.11) for CNS tumors. This suggests that background ionizing radiation contributed to the risk of leukemia and CNS tumors in children.^30^

Meadows et al. analyzed the development of secondary malignancies in pediatric cancer survivors treated with radiation therapy, finding a substantial risk of new cancers resulting from therapeutic radiation exposure.^31^

Nuclear accidents, such as the Chernobyl disaster in 1986, have provided critical data on the effects of ionizing radiation on human populations. Children exposed to radiation released by the accident had a significant increase in the incidence of thyroid cancer.^32^

Non-ionizing radiation does not have enough energy to ionize atoms or molecules, that is, it cannot remove electrons from atomic structures. Although it does not cause ionization, it can still cause other biological effects, such as the heating of tissues. The main sources are ultraviolet light, electromagnetic fields, and radiation from electronic devices.^1^^,^^2^

Epidemiological evidence on the relationship between non-ionizing radiation and pediatric cancer varies in strength, and there is limited scientific consensus on the exact risks.

Some epidemiological studies suggest a possible association between exposure to low-frequency electromagnetic fields (LFEM) and an increased risk of childhood leukemia. For example, a review study by Kheifets et al. found a statistically significant association between high exposure to LFEM and the risk of leukemia in children.^33^

However, other studies and reviews, such as the one conducted by the International Commission on Non-Ionizing Radiation Protection (ICNIRP), have not found conclusive evidence that LFEM causes cancer in children.^34^

Exposure to radiofrequency (RF), especially from cell phones, has been studied for the risk of brain tumors in children and adolescents. A multicenter study found no significant increase in the risk of brain tumors in regular cell phone users among children and adolescents.^35^

Exposure to ultraviolet (UV) radiation, especially UVB, is a well-established risk factor for skin cancer. In children, intense sun exposure during childhood may increase the risk of developing melanoma in adulthood. Preventive measures, such as the use of sunscreen and protective clothing, are recommended to minimize the risks.^36^ The relationship between non-ionizing radiation and pediatric cancer is complex and controversial. The main controversies include inconsistencies in the results of epidemiological studies and limitations in exposure methods.

The difficulty in accurately measuring individual exposure to non-ionizing radiation prevents an adequate interpretation of the results. Variability in exposure sources and radiation levels makes comparative studies challenging.

## Soil and food contaminants

The introduction to the article “Carcinogens and Anticarcinogens in the Human Diet: A Comparison of Naturally Occurring and Synthetic Substances” by the National Research Council Committee on Comparative Toxicity of Naturally Occurring Carcinogens states that each one of us establishes our own concepts of risk when crossing the street, traveling by plane, or learning about potential threats to our health and well-being. Risks associated with the possible presence of carcinogens in the air we breathe, the water we drink, or the food we eat evoke a strong emotional response, often questioning the source of the information and prompting correction of the situation. The large influx of published articles has saturated people's ability to differentiate the important from the trivial and to discriminate facts from hypotheses.^37^ For this reason, it is essential to take a critical look at the source of the publications.

The authors will cite some information with greater evidence, such as that from a group of researchers from the United States of America and Toronto who carried out a systematic review and meta-analysis of prospective studies, to summarize the evidence of the association of red meat and processed meat with cancer. High consumption of red meat was associated with a higher risk of breast, endometrial, colorectal, colon, rectal, lung and liver cancer. Processed meat consumption was associated with a 6% higher risk of breast cancer, 18% of colorectal cancer, 21% of colon cancer, 22% of rectal cancer and 12% of lung cancer.^38^

In this context, INCA recommends the consumption of red meat for up to 500 grams of cooked meat per week. The way meat is prepared is also important to prevent cancer, and the best meats are roasted, boiled and stewed.^39^

Soil contaminants can compromise food at several stages of the food chain, whether in primary production, processing, or distribution. Endocrine disruptors may be present and act in very low doses, interfering with the production, release and elimination of hormones. These include dioxins, furans, polychlorinated biphenyls, various solvents, heavy metals, pesticides, cosmetics, plastics, and numerous chemicals.^40^

In rodents, there is evidence that some chemicals that occur naturally in the diet may be carcinogenic, including those derived from food preparation such as heterocyclic amines generated during cooking and nitrosamines, and agents produced during food storage such as aflatoxins and other fungal toxins, which have been associated with liver cancer.^37^

Soil contamination, whether by naturally occurring agents or resulting from human activities, can pose an increased risk of cancer in three distinct ways: by inhalation, as is the case with contamination by asbestos or other mineral fibers that, when inhaled, can cause lung cancer; accidental ingestion, especially by children playing in contact with the soil; or through contamination of the food chain, surface water or groundwater.^2^

Industrial activities account for two-thirds of contaminated areas, with the most common contaminants being heavy metals, mineral oils and aromatic hydrocarbons. Estimates of the risk of cancer associated with such contaminants are available in only a few countries. In Italy, an epidemiological surveillance project in contaminated areas found a higher incidence of cancer of 9% in men and 7% in women.^2^

Dioxins originate from the incomplete combustion of waste and from foundries and steel industries and are considered group I carcinogens in the IARC classification.^38^

Lindane is a halogenated aromatic hydrocarbon with insecticidal properties. It is used in fruit and vegetable crops and in baits and seed treatments for rodent control. Using the IARC registry of workers exposed to phenoxy herbicides and their contaminants, Kogevinas et al. found a higher risk of developing non-Hodgkin's lymphoma in workers exposed to lindane, with an OR of 1.6 (CI 0.3–8.8).^40^

Other pesticides that also contaminate the soil are classified as probably carcinogenic (group 2A): the fungicide captafol, DDT, malathion, diazinon, dieldrin, the fumigant ethylene dibromide and the herbicide glyphosate.^2^

Pesticides with carcinogenic risk are registered, or in use, in 62% of the countries worldwide. The Brazilian population consumes 20% of the total pesticides in the world, which represents 300,000 tons per year. It is estimated that in the last 40 years, while Brazilian agriculture increased by only 78%, pesticide consumption increased by 700%.^38^ Panis et al.^41^ observed that contamination of drinking water, when analyzed for 11 pesticides, was significantly associated with the estimated number of cancer cases in Paraná, Brazil (R = 0.58 and *p* < 0.0001).

## Conclusions

Given that between 30% and 50% of adult cancers are preventable, it is important to implement evidence-based strategies for the prevention, early detection and treatment of people with the disease.

Compared to adults, children are more vulnerable to environmental agents due to their unique activity, behavior and physiology patterns, as well as their organ immaturity. Moreover, many children, especially those living in low-income regions of the world, are engaged in hazardous work, such as that involving contact with pesticides, and are exposed to emerging threats, such as toxic components of electronic waste.^2^

Preventing environmental exposure to carcinogenic pollutants stems primarily from uncontrolled urbanization and industrialization and requires both government regulation and community action and commitment. The issue of work-related cancer demands priorities focused on social determination and action in multiple spheres, beyond health, with the ultimate goal of preventing exposure.^24^

In this scenario, strategies to address the issue should include significant investments in public infrastructure for water quality, wastewater monitoring, sanitation and hygiene, replacement of lead service lines, and well water improvement programs. Actions should also be taken to protect the population from harmful exposures to toxic substances, including new and existing chemicals, with special attention paid to children, pregnant women, and other susceptible populations.^42^

Additionally, sustainable and sufficient funding for federal agencies with environmental health missions is important; efforts to reduce indoor and outdoor air pollution by setting robust air quality standards for ozone, particulate matter, nitrogen dioxide, carbon monoxide, and other pollutants to protect public health and well-being; government education campaigns on cancer risk factors, such as sun exposure and tobacco use; and comprehensive action to achieve environmental justice.^42^

## Conflicts of interest

The authors declare no conflicts of interest.
